# Supplementary material for: Collective predator evasion: Putting the criticality hypothesis to the test
Source: PLoS Comput Biol. 2021 Mar 15;17(3):e1008832. doi: 10.1371/journal.pcbi.1008832 (PMC7993868; doi:10.1371/journal.pcbi.1008832)
Supplement: S1 Text — Sections: I. Model-Description. II. Model parameters. III. Evolutionary algorithm and ESS. IV. Measures of self-sorting. V. Susceptibility under a homogeneous global field. VI. Balancing social vs. direct predator information. VII. Robustness against modifications of the prey & predator dynamics and the selection mechanism. (PDF) [file pcbi.1008832.s001.pdf]

SI Appendix
Supplementary Information
"Collective predator evasion: Putting the criticality hypothesis to the test"

Pascal P. Klamser[1,2], Pawel Romanczuk[1,2*]

1 Department of Biology, Institute for Theoretical Biology, HumboldtUniversität zu Berlin, 10115 Berlin, Germany
2 Bernstein Center for Computational Neuroscience, 10115 Berlin, Germany

I Model-Description

I.1 Prey-Agents

The prey agents are modeled as active Brownian particles with constant speed $v = v_0$ and angular noise [1]. The stochastic equations of motion read:

$$\frac{d\vec{r}_i(t)}{dt} = \vec{v}_i(t) \tag{S1a}$$

$$\frac{d\varphi_i(t)}{dt} = \frac{1}{v_0}\left(F_{i,\perp}(t) + \sqrt{2D}\xi(t)\right) , \tag{S1b}$$

with $F_{i,\perp}(t) = \vec{F}_i(t) \cdot \vec{e}_{i,\perp}$ being the force acting on agent i projected on the direction perpendicular to the direction of motion $\vec{e}_{i,\perp}$, D being the angular diffusion coefficient and $\xi(t)$ being Gaussian white noise with zero mean and vanishing temporal correlations. For simplicity we omit in the following the explicit time dependence of positions, velocities and forces.

Agents react to their environment by (i) coordinating their direction of motion with their neighbors through an alignment interaction, (ii) by trying to maintain a preferred distance to conspecifics (long-ranged attraction and short-ranged repulsion) and (iii) by a fleeing response (repulsion) from the predator. The alignment force between a focal agent i and all its neighbors $j \in \mathbb{N}_i$

$$\vec{F}_{i,a} = \frac{1}{|\mathbb{N}_i|} \sum_{j \in \mathbb{N}_i} \mu_{alg} \cdot \vec{v}_{ji}. \tag{S2}$$

acts towards minimizing the velocity difference $\vec{v}_{ji} = \vec{v}_j - \vec{v}_i$ with the alignment strength μ_{alg}.

Individuals attempt to maintain a preferred distance r_d to each other through a distance regulating force

$$\vec{F}_{i,d} = \frac{1}{|\mathbb{N}_i|} \sum_{j \in \mathbb{N}_i} \mu_d \cdot \tanh\left(m_d(r_{ji} - r_d)\right) \cdot \hat{r}_{ji} \tag{S3}$$

with $\hat{r}_{ji} = \frac{\vec{r}_j - \vec{r}_i}{|\vec{r}_j - \vec{r}_i|}$ being the unit vector along the distance vector from agent i to j, μ_d as strength of the force and m_d as the steepness of the change from repulsion (for $r_{ji} < r_d$) to attraction (for $r_{ji} > r_d$), as illustrated in Fig. S1A. Finally if a predator p is a neighbor of agent i, $p \in \mathbb{N}_i$, the agent is repelled with

$$\vec{F}_{i,f} = -\mu_{flee} \cdot \hat{r}_{pi} \tag{S4}$$

otherwise $\vec{F}_{i,f} = 0$. The total force governing the movement decision of agent i is defined as

$$\vec{F}_i = \vec{F}_{i,d} + \vec{F}_{i,alg} + \vec{F}_{i,flee} \ . \tag{S5}$$

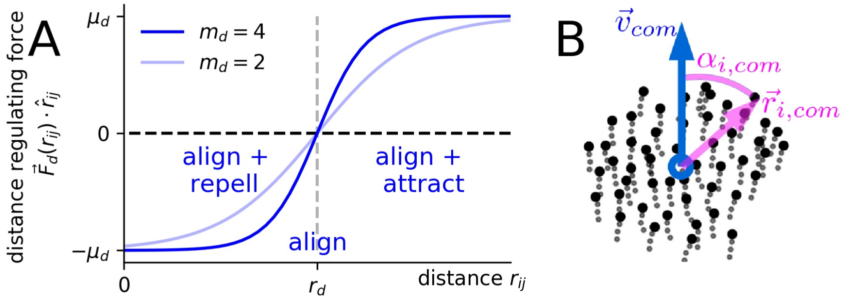

Figure S1: **Illustration of the distance regulating force.** **A**: Distance regulating force $\vec{F}_d(r_{ij})$ between agents i and j projected on the separation direction $\hat{r}_{ji} = \frac{\vec{r}_j - \vec{r}_i}{|\vec{r}_j - \vec{r}_i|}$. The force equals zero at the preferred distance $r_d = 1$ and is displayed for a distance regulating force steepness $m_d = 2$ (used in the simulations) and $m_d = 4$. **B**: Relative polar coordinates of an agent i with respect to the center of mass $\vec{r}_{com}$ of the school (blue circle) and to the average velocity of the school $\vec{v}_{com}$ (blue arrow). The angle $\alpha_{i,com}$ (magenta arc) between the school velocity and the agents i current position $\vec{r}_{i,com}$ (magenta arrow) and the distance to the center of mass $|\vec{r}_{i,com}|$ define the position in this relative coordinate system.

I.2 Predator-Agent

For simplicity the predator obeys a deterministic equation of motion for the heading angle, analogous to Eq. S1b but without the angular noise term:

$$\frac{d\varphi_p}{dt} = \frac{1}{v_p} \vec{e}_{p,\perp} \cdot \vec{F}_p \ . \tag{S6}$$

Here, v_p is the fixed predator speed and $\vec{F}_p$ is the predator pursuit force. In this study we consider a predator faster than the prey $v_p > v_0$. We assume that the predator can only attack one prey at a time. It considers prey individuals which are its frontal Voronoi-neighbors $\mathbb{N}_p$ as targets and selects equally likely among them:

$$p_{select,i} = \begin{cases} \frac{1}{|\mathbb{N}_p|} & \text{if } i \in \mathbb{N}_p \\ 0 & \text{otherwise .} \end{cases} \tag{S7}$$

The limitation of potential targets to its frontal Voronoi-neighbors $\mathbb{N}_p$, is motivated by kinematic and sensory constraints of the predator. If the predator launches an attack, with an attack rate γ_a, which also accounts for potential handling time, it's success probability is linearly dependent on distance and vanishes at distances larger than r_{catch}:

$$p_{success,i} = \begin{cases} \frac{r_{catch} - r_{ip}}{r_{catch}} & \text{if } r_{ip} < r_{catch} \\ 0 & \text{otherwise.} \end{cases} \tag{S8}$$

In summary, the probability that a predator successfully catches a targeted agent within a small time window $[t, t + \delta t]$ is

$$p_{catch,i}(t, \delta t) = p_{success,i}(t) \cdot p_{select,i}(t) \cdot \gamma_a \delta t \ . \tag{S9}$$

The predators movement is biased towards the weighted center of mass of the prey school, where each prey position is weighted by its probability of a successful catch $p_{catch,i}(t, \delta t)$. Since $p_{catch,i}$ is non-zero only for the predator's frontal Voronoi-neighbors, the predator movement are governed by local, visually accessible information. The pursuit force is thus

$$F_p = \mu_p \cdot \left(\sum_i p_{catch,i} \vec{r}_{ip} \right) \ . \tag{S10}$$

II Model parameter

The default model parameters used are listed in Tab. S1. Note that two parameters can be eliminated by rendering the equations dimensionless. If, for instance, the preferred distance r_d and the prey speed v_0 are used to define the

	parameter	symbol	value
prey	angular diffusion	D	0.5
	alignment strength	μ_{alg}	evolves
	distance strength	μ_d	2
	distance steepness	m_d	2
	(distance preferred)	r_d	1
	(speed)	v_0	1
	flee strength	μ_{flee}	4
predator	speed	v_p	2
	pursuit strength	μ_p	2
	attack rate	γ_a	1/3
	catch radius	r_{catch}	3
simul.	number of agents	N	400
	time step	dt	0.02
	equilibration time	T_{eq}	200
	simulation time	T_{simu}	120
	mutation rate	γ_m	0.8
	mutation strength	σ_m	0.075

Table S1: **Default model parameters used.** Time and space have been rescaled to dimensionless units by setting, without loss of generality, the prey speed v_0 and preferred distance r_d to 1. All length scales are thus measured in units of r_d, and all time scales in terms of time needed to move the distance r_d. Note that the flee strength μ_{flee} is strictly speaking a predator-prey parameter which reduces the prey-only parameters to four.

characteristic length L and time T:

$$L = r_d,\ T = \frac{r_d}{v_0}, \tag{S11}$$

the Eq. S1 can be reformulated to

$$\frac{d\vec{r'}_i}{dt'} = \vec{v'}_i \tag{S12a}$$

$$\frac{d\varphi_i}{dt'} = \frac{r_d}{v_0^2}\left(F_{i,\perp} + \sqrt{2D}\sqrt{\frac{v_0}{r_d}}\xi(t')\right) \tag{S12b}$$

$$= F'_{i,\perp} + \sqrt{\frac{2D_{rot}r_d}{v_0}}\xi(t'). \tag{S12c}$$

Here is $D_{rot} = \frac{D}{v_0^2}$ the rotational diffusion coefficient (with the unit $[D] = 1/t$). The primed variables are the dimensionless counterparts

$$t = \frac{r_d}{v_0}t',\ v_i = v_0 v'_i,\ r_i = r_d r'_i \tag{S13}$$

and note that the Gaussian stochastic process is transformed according to

$$\xi(t) = \sqrt{\frac{v_0}{r_d}}\xi(t'). \tag{S14}$$

With this choice of characteristic length and time and setting $v_0 = 1$ and $r_d = 1$, the dimensionless parameters keep their values listed in Tab. S1.

Since the flee strength μ_{flee} is a predator-prey interaction parameter, the prey system has effectively only four parameters from which the alignment strength μ_{alg} is evolving. The remaining prey parameters are the angular-diffusion coefficient D which is set to $D = 0.5$ resulting in a persistence time of $\tau_p = \frac{v_0^2}{D} = 2$, i.e. a solitary agents maintains it current direction of motion for approximately the distance of two body length. The distance regulating strength $\mu_d = 2$ is chosen to ensures that the prey group stays cohesive. The distance steepness $m_d = 2$ regulates how quick the distance regulating force saturates to its maximal/minimal values at distances below or above the preferred distance r_d (Fig. S1A).

For the predator the speed must be larger than the prey-speed and is set to $v_p = 2$. Its pursuit strength μ_p describes together with the speed its turning ability and is set to $\mu_p = 2$ and therefore equals the preys distance regulating force

strength. With an capture rate $\gamma_c = 1/3$ and a simulation time of $T = 120$ around forty prey are captured per round which corresponds to 10% of the entire school. The catch radius is set to $r_{catch} = 3$ and therefore corresponds to three body length.

The simulation parameters, and in particular the shoal-size of $N = 400$, have been chosen in order to simulate biologically reasonable behavior, while at the same time limiting the computational costs. For each generation of the evolutionary simulations, 76 independent runs are performed, with each equilibrating for $T_{eq} = 200$ before the predator appears, and then running for $T_{simu} = 120$ time units. The time-step is set to $dt = 0.02$ which provides sufficient numerical stability and efficient computation (see sectionII.1).

II.1 Numeric stability

This section addresses the numerical stability of the Euler-Maruyama method used to simulate the stochastic differential equations. The time-step dt should be much smaller than the persistence time $\tau_p = 2$, smaller than the shortest correlation time, small enough to fulfill the stability criterion and to avoid oscillating behavior. An even stricter criterion is that the time step is smaller than a $1/10$ of the correlation time of the fastest process

$$\frac{1}{10|\mu|} \leq dt. \tag{S15}$$

Here μ is the strength of the strongest force (e.g. alignment-, flee-, repulsion-force).

III Evolutionary algorithm and ESS

The evolutionary algorithm is designed to mimic natural selection at the level of behavioral phenotypes. Among others, the influence of fecundity selection or sexual selection is neglected and the fitness function is only based on how likely an individual is captured in a predator attack, which is a biologically reasonable simplification in the context of predator-prey interactions. The algorithm consists of (i) a fitness estimation step, (ii) a fitness-proportionate-selection step and (iii) a mutation step.

(i) The fitness is estimated by running $N_f = 76$ independent attack-simulations on the same phenotype population. For each simulation the $\gamma_a \cdot T_{simu}$ agents with the highest cumulative probability of capture (Eq. S9) are declared as dead. The fitness of agent i is:

$$f_i = -N_{c,i} + max(N_{c,j}, j). \tag{S16}$$

Here $N_{c,i}$ is the number of simulations in which agent i was captured and $max(N_{c,j}, j)$ is the largest number of deaths among all agents.

(ii) The N offspring are generated via the fitness-proportionate-selection. Thereby has one offspring the parameters of the parent i with probability

$$p_{parent,i} = \frac{f_i}{\sum_j f_j}. \tag{S17}$$

(iii) An offspring agent mutates with a probability γ_m, the mutation rate, by adding to its alignment strength μ_{alg} a Gaussian random variable with zero mean and standard deviation σ_m, as the mutation strength.

Steps (i) till (iii) are repeated in each generation.

Note that instead of step (i) the agents could directly get captured during the simulation and removed from the group during the run. This however introduces an additional source of noise in the predation process and the resulting fitness gradient of the prey would become more noisy. As a consequence the number of generations needed to reach an ESS increases. Nevertheless, to ensure the robustness of our results we repeated the evolution with captures during the evolution, which did not change the final results (see Sect. VII).

III.1 Estimation of the evolutionary stable state (ESS)

In the evolutionary algorithm the finite mutation strength and the stochastic roulette-wheel selection introduce noise on top of the intrinsic stochasticity of the the predator-prey dynamics (Eq. S1). This stochasticity is essential for evolutionary adaptation and exploration of the phenotype space, but makes it challenging to identify the evolutionary stable states (ESS) with high precision in evolutionary simulations.

To circumvent this uncertainty about the exact optimum, we estimate the evolutionary stable state based on the zero-crossing of the fitness-gradient estimated from numerical simulations. For a system in generation g with agent parameters $\vec{\mu}_{alg}(g) \in \mathbb{R}_+^N$ the estimated fitness gradient $\nabla f(g)$ is computed by predicting the mean outcome of the fitness-proportionate selection

$$\langle \mu_{alg} \rangle_{predict}(g) = \vec{p}_{parent,i} \cdot \vec{\mu}_{alg} \tag{S18a}$$

$$= \frac{1}{\sum_j^N f_j} \sum_i^N f_i \mu_{alg,i} \tag{S18b}$$

and subtracting from it the current mean-value:

$$\nabla f(g) = \langle \mu_{alg} \rangle_{predict} - \langle \mu_{alg} \rangle. \tag{S19}$$

Note that, in sake of readability, we omitted for terms on the RHS of Eqs. S18, S19 the dependency on the generation g.

The average fitness gradient corresponding to an alignment strength is

$$\nabla f(\mu_{alg}, \Delta_\mu) = \langle \nabla f \rangle_{\mathbb{S}_{\mu_{alg},\Delta_\mu}} = \frac{\sum_{g \in \mathbb{S}_{\mu_{alg},\Delta_\mu}} \nabla f(g)}{|\mathbb{S}_{\mu_{alg},\Delta_\mu}|} \tag{S20}$$

where $\mathbb{S}_{\mu_{alg},\Delta_\mu}$ is the set of generations which fulfill the condition:

$$\mu_{alg} - \Delta_\mu/2 \leq \langle \mu_{alg} \rangle(g) \leq \mu_{alg} - \Delta_\mu/2. \tag{S21}$$

Therefore, Eq. S20 represents a simple binning of generations with a bin-width of Δ_μ. The maximum of the estimated fitness landscape, i.e. the evolutionary stable state, is where the estimated fitness gradient is zero and where its slope is negative. An detail illustration of all components needed to compute the ESS as proposed here is shown in Fig. S2.

IV Measures of self-sorting

Here we explain in detail the relative positions of individuals in the swarm with respect to the front-back, side-center dimensions and local density.

IV.1 Relative positions

In order to define the relative positions with respect to the front-back and to the side-center dimensions, we first represent every agent position by its distance to the center of mass of the collective

$$r_{i,com} = |\vec{r}_{i,com}| = \vec{r}_i - \vec{r}_{com} \text{ with } \vec{r}_{com} = \sum_i \vec{r}_i / N \tag{S22}$$

and the angle between its position and the mean velocity of the collective

$$\alpha_{i,com} = \angle(\vec{r}_{i,com}, \vec{v}_{com}) \text{ with } \vec{v}_{com} = \sum_i \vec{v}_i / N\ . \tag{S23}$$

We refer to this representation as the *relative polar coordinates*, illustrated in Fig. S1B. Note that the x-axis is parallel to $\vec{v}_{com}$, the center of mass is at the origin and the quadrants IV and III are folded onto I and II respectively. The folding is reasonable if a left-right symmetry holds, which we assume. The relative front position is

$$\tilde{r}_{i,f} = r_{i,com} \cos \alpha_{i,com} \tag{S24}$$

with its normalized version as

$$r_{i,f} = \frac{\tilde{r}_{i,f} - \min(\tilde{r}_{j,f}, j)}{\max(\tilde{r}_{j,f}, j) - \min(\tilde{r}_{j,f}, j)} \tag{S25}$$

which results in front positions in the interval $r_{i,f} \in [0, 1]$, with 0 corresponding to individuals at the very rear of the school and 1 to individuals at the very front.

The relative side-position is

$$\tilde{r}_{i,s} = r_{i,com} \sin \alpha_{i,com} \tag{S26}$$

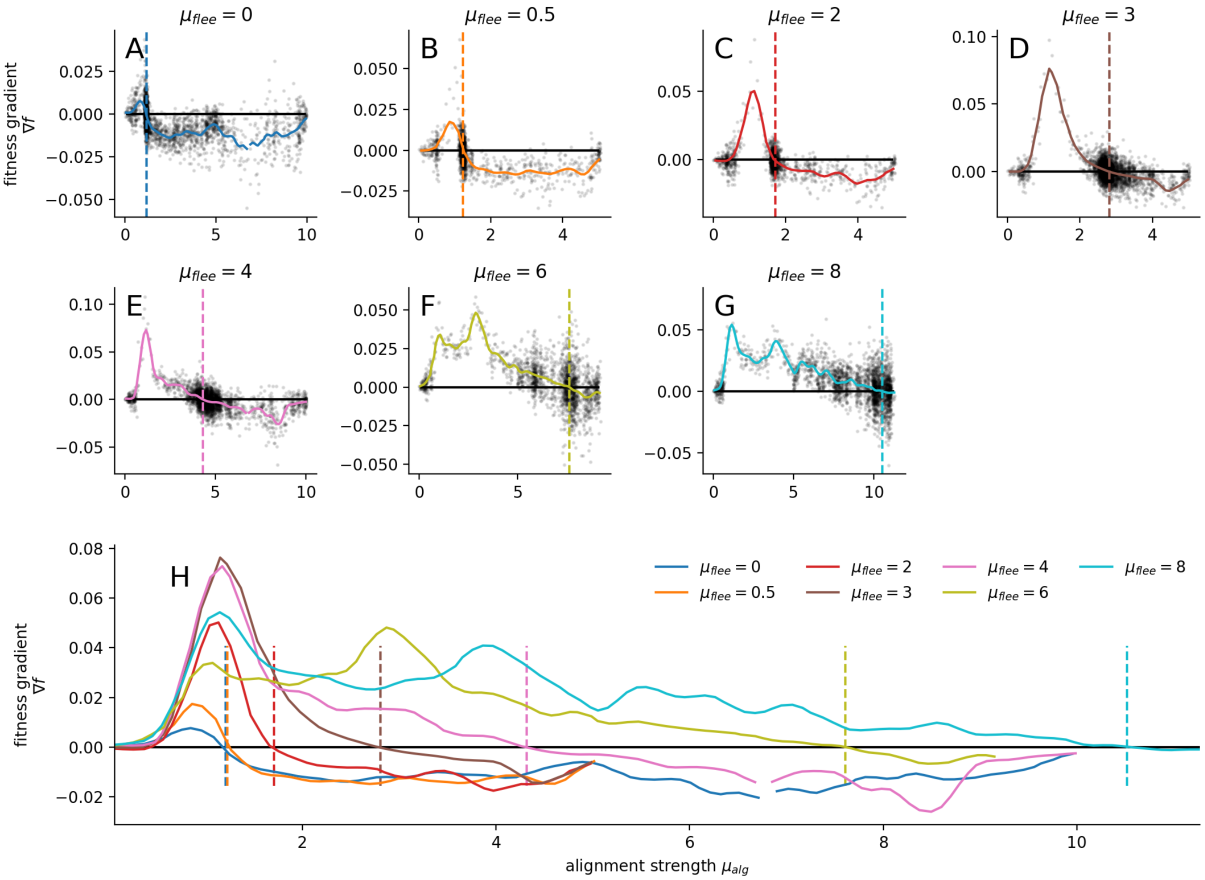

Figure S2: **Fitness gradients for different flee-strength to estimate the ESSs.** Details on the estimation of evolutionary stable states of Fig. 4 in the main text. **A** - **G**: Fitness gradient ∇f for evolution with different flee strength μ_{flee}. Black-dots indicate the estimated fitness gradients for each generation. Solid lines are averaged fitness gradients. Dashed vertical lines indicate where $\nabla f = 0$ and thus mark the evolutionary stable states. **H**: All fitness gradients displayed together. Note that the peaks for $\mu_{flee} = 6$ at $\mu_{alg} \approx 3$ and for $\mu_{flee} = 8$ at $\mu_{alg} \approx 4$ are due to fluctuations in the standard-deviation of the population. If the standard-deviation is kept constant those peaks vanish (not shown).

with its normalized version as

$$r_{i,s} = \tilde{r}_{i,s} / \max(\tilde{r}_{j,s}, j) \; . \tag{S27}$$

We apply the normalization because we are interested if an individual is at the front and not how far the front is away from the center of mass. As a results, the normalized measures are less noisy if we average over independent initializations. The average normalized relative-position over S samples is

$$\langle r_{i,x} \rangle = \frac{\sum_{k=1}^{S} r_{i,x,k}}{S} \tag{S28}$$

with $r_{i,x,k}$ as the normalized relative position of agent i in the kth sample run. Note that the normalized relative position is computed after the equilibration time T_{eq}.

IV.2 Local density

The local density of agent i is computed through its distance to the kth nearest neighbor $d_{i,kN}$ to

$$\rho_i = k / A(d_{i,kN}, d_{i,e}) \; . \tag{S29}$$

The term $A(d_{i,kN}, d_{i,e})$ represents the corrected area. If the agents distance to the edge of the collective $d_{i,e}$ is larger as $d_{i,kN}$, no correction is needed and the area is the area of a circle with radius $d_{i,kN}$. If the distance to the edge is smaller than $d_{i,kN}$, the circle-area is corrected by subtracting the area of the circle segment with a sagitta (height) of $h = d_{i,kN} - d_{i,e}$. Therefore, the area is computed as

$$A(d_{i,kN}, d_{i,e}) = \begin{cases} \Phi d_{i,kN}^2 & \text{if } d_{i,kN} < d_{i,e} \\ \Phi d_{i,kN}^2 - d_{i,kN}\left(d_{i,kN} \arccos \frac{d_{i,e}}{d_{i,kN}} - d_{i,e}\sqrt{1 - \frac{d_{i,e}^2}{d_{i,kN}^2}}\right) & \text{otherwise.} \end{cases} \quad \text{(S30)}$$

This correction is good if the edge of the collective has a small local curvature compared to the curvature of the circle with radius $d_{i,kN}$. This should be fulfilled because a collective of $N = 400$ individuals with a preferred distance of $r_d = 1$ and a spherical form has a radius of $R \approx 11$ while the distance to the kth nearest neighbor with $k = 10$ and a Voronoi-interaction network is between 1 and 2.

IV.3 Assortativity

The assortativity r is defined as

$$r = \frac{1}{\sigma_q^2} \sum_{j,k} jk(e_{j,k} - q_j q_k) \quad \text{(S31)}$$

with $e_{i,j}$ as the joint probability that a randomly drawn edge connects vertices of type i and j, and q_x is the probability that a node of type x is at one end of a randomly drawn edge, i.e. it is the fraction of edges that have a vertex of type x at one end. The assortativity is the Pearson correlation coefficient over the values of the vertices connected by edges.

V Susceptibility under a homogeneous global field

The susceptibility is in general defined by how strong a macroscopic observable $\langle m \rangle$ changes if an external field h is changed

$$\chi = \frac{\partial \langle m \rangle}{\partial h} . \quad \text{(S32)}$$

In the Ising-model, the susceptibility defined in Eq. S32 describes the change of the magnetization per spin

$$m = \frac{M}{N} = \frac{1}{N} \sum_{i=1}^{N} s_i , \quad \text{(S33)}$$

given the change of an external field h The s_i is the spin at side i which can be either up or down, i.e. $s_i \in [-1, 1]$. Interestingly, the response to a (weak) field can be linked to fluctuations in the order parameter in the absence of a field [2]. In statistical physics the probability to observe the system in the state $\vec{s} = [s_0, s_1, \ldots, s_N]$ is

$$P(\vec{s}) = \frac{\exp[-\beta H(\vec{s})]}{Z} . \quad \text{(S34)}$$

$H(\vec{s})$ describes the energy of the system at state $\vec{s}$ and β is the inverse of the thermal energy $\beta = 1/(k_b T)$ with k_b as the Boltzmann constant and T as the temperature of the surrounding heat bath. Thus, the state $\vec{s}$ is more likely the smaller its corresponding energy. The partition function

$$Z = \sum_{\{\vec{s}\}} \exp[-\beta H(\vec{s})] \quad \text{(S35)}$$

normalizes the probability with $\sum_{\{\vec{s}\}}$ as a sum over all possible system states. If spins tend to align with the external field, the energy is partly defined as $H(s_i) = ... - h \sum_i s_i$. Now, the mean magnetization per spin can be computed to

$$\langle m \rangle = \sum_{\{\vec{s}\}} m(\vec{s}) P(\vec{s}) = 1/N \frac{1}{\beta} \frac{\partial \ln Z}{\partial h} . \quad \text{(S36)}$$

This allows us to derive the susceptibility χ defined in Eq. S32 to

$$\chi = \frac{1}{\beta} \frac{\partial^2 \ln Z}{\partial h^2} = \frac{\beta}{N} [\langle M^2 \rangle - \langle M \rangle^2] = \beta N [\langle m^2 \rangle - \langle m \rangle^2] \quad \text{(S37)}$$

The above relation connects the response of the system to an infinitesimally small change of the external field h with fluctuations in the order parameter. The linear nature of this response to small changes can also be assessed by a Taylor-expansion to linear order of the canonical distribution around $h = 0$ (see for example Eq. 1.21 in [2]). The response can be reformulated to highlight the link to the connected spin correlation function or spin pair correlation function

$$\chi = N\beta[\langle m^2\rangle - \langle m\rangle^2] = \frac{\beta}{N}\left[\left\langle \sum_{ij} s_i s_j \right\rangle - \left\langle \sum_i s_i \right\rangle \cdot \left\langle \sum_j s_j \right\rangle\right] \tag{S38a}$$

$$= \frac{\beta}{N}\sum_{ij}[\langle s_i s_j\rangle - \langle s_i\rangle\langle s_j\rangle]. \tag{S38b}$$

In the following, we establish an analog description for the model system (presented in Sect. I) with fixed speed.

V.1 Susceptibility of the prey collective in equilibrium

For simplicity we assume, as in the section before, that the prey agents (Sect. I) react to a global homogeneous field $\vec{h}$. From Eq. S1 the change in heading of individual i in response to $\vec{h}$ is

$$\frac{d\varphi_i}{dt} = \frac{\vec{h}\hat{e}_{\varphi,i}}{v_0} = F_{i,s} \text{ with } \hat{e}_{\varphi,i} = [-\sin\varphi_i, \cos\varphi_i]\ . \tag{S39}$$

From this force $F_{i,s}$ the analog to energy $H_{s,i}$ for individual i can be computed via integration to

$$H_{s,i} = -\frac{\vec{h}\hat{u}_i}{v_0} \text{ with } \hat{u}_i = [\cos\varphi_i, \sin\varphi_i]\ . \tag{S40}$$

The total energy is composed of the sum of isolated components $H_{s,i}$ and of the part that is influenced by the interactions in between the prey H_m:

$$H = H_m(\vec{\varphi}) + \sum_i H_{s,i}(\varphi_i, \vec{h}) = H_m(\vec{\varphi}) + -\frac{\vec{h}}{v_0}\cdot\sum_i \hat{u}_i \tag{S41}$$

with $\vec{\varphi} = [\varphi_0, \varphi_1, \ldots, \varphi_N]$. Only $H_{s,i}$ depends on the external field $\vec{h}$. Knowing the energy of the systems allows (analog to Eq. S34) to define a probability to observe the state $\vec{\varphi}$ which is

$$P(\vec{\varphi}) = c_H\frac{\exp[\beta\vec{h}\sum\hat{u}_i]}{Z} = c_H\frac{\exp[\beta N\vec{h}\vec{\phi}]}{Z} \tag{S42}$$

with $c_H = e^{-\beta H_m}$. However, note that Eq. S34 assumes that there is a heat bath represented by $\beta = 1/(k_b T)$. Since the strength of the angular noise D (see Eq. S1) can prevent polarization in the prey collective, it plays a similar role as the temperature in the Ising model. Therefore, we use $\beta = 1/(Dv_0)$ to compute the expectation value of the polarization vector $\vec{\Phi} = \frac{1}{N}\sum_i^N \hat{u}_i$ (analog to the computation of the mean magnetization in the Ising model).

$$\left\langle\vec{\Phi}\right\rangle = \sum_{\{\vec{\varphi}\}}\vec{\Phi}P(\vec{\varphi}) = \frac{1}{N\beta}\vec{\nabla}_{\vec{h}}\ln Z \tag{S43a}$$

$$= \frac{1}{N\beta}\begin{pmatrix}\frac{\partial}{\partial h_x}\\ \frac{\partial}{\partial h_y}\end{pmatrix}\ln\left(\sum_{\{r,\varphi\}} c_H e^{\beta\vec{h}\cdot\vec{M}}\right)\ , \tag{S43b}$$

with $\vec{M} = N\vec{\Phi}$. Finally, we compute the susceptibility as the sum of changes of the polarization vector $\left\langle\vec{\Phi}\right\rangle$ components with respect to the external field $\vec{h}$. It can be written more compact with the $\vec{h}$-Laplace operator $\Delta_{\vec{h}} = \frac{\partial^2}{\partial h_x^2} + \frac{\partial^2}{\partial h_y^2}$ to

$$\chi = \vec{\nabla}_{\vec{h}}\left\langle\vec{\Phi}\right\rangle = \frac{1}{N\beta}\Delta_{\vec{h}}\ln(Z) \tag{S44a}$$

$$= \frac{\beta}{N}\left[\langle M_x^2 + M_y^2\rangle - \left(\langle M_x\rangle^2 + \langle M_y\rangle^2\right)\right] \tag{S44b}$$

$$= \frac{\beta}{N}\left[\left\langle\vec{M}\cdot\vec{M}\right\rangle - \left\langle\vec{M}\right\rangle\cdot\left\langle\vec{M}\right\rangle\right] \tag{S44c}$$

$$= \beta N\left[\left\langle\vec{\Phi}\cdot\vec{\Phi}\right\rangle - \left\langle\vec{\Phi}\right\rangle\cdot\left\langle\vec{\Phi}\right\rangle\right] = \beta N\left[\langle\Phi^2\rangle - \langle\Phi\rangle^2\right]\ . \tag{S44d}$$

This is analogous to Eq. S38 and establishes a link to the pair-correlation between individual heading direction. Analogously to Eq. S38, we may also write:

$$\chi = N\beta \left[\left\langle \vec{\Phi} \cdot \vec{\Phi} \right\rangle - \left\langle \vec{\Phi} \right\rangle \cdot \left\langle \vec{\Phi} \right\rangle \right] \tag{S45a}$$

$$= \frac{\beta}{N} \left[\left\langle \sum_i \hat{u}_i \cdot \sum_j \hat{u}_j \right\rangle - N^2 \left\langle \vec{\Phi} \right\rangle \cdot \left\langle \vec{\Phi} \right\rangle \right] \tag{S45b}$$

$$= \frac{\beta}{N} \left[\left\langle \sum_{ij} \hat{u}_i \cdot \hat{u}_j \right\rangle - \sum_{ij} \left\langle \vec{\Phi} \right\rangle \cdot \left\langle \vec{\Phi} \right\rangle \right] \tag{S45c}$$

$$= \frac{\beta}{N} \sum_{ij} \left[\langle \hat{u}_i \cdot \hat{u}_j \rangle - \left\langle \vec{\Phi} \right\rangle \cdot \left\langle \vec{\Phi} \right\rangle \right] \tag{S45d}$$

$$= \frac{\beta}{N} \sum_{ij} \left\langle \left(\hat{u}_i - \left\langle \vec{\Phi} \right\rangle \right) \cdot \left(\hat{u}_j - \left\langle \vec{\Phi} \right\rangle \right) \right\rangle . \tag{S45e}$$

Note that the above derivation until Eq. S44 assumes a thermodynamic equilibrium and is for the out-of-equilibrium prey model strictly speaking not valid (see [3, 2] for discussion of non-equilibrium approaches). However, from Eq. S44 to Eq. S45 there is no such assumption. It is merely a reformulation and therefore valid. It means, we can interpret χ always as the sum over the correlation in velocity fluctuations over all pairs. In other words, the larger χ the stronger is the mean correlation of directional information between random pairs.

V.2 Difference between susceptibility and predator response

We assumed in Sect.V that (i) the system is in thermodynamic equilibrium (ii) the changes of the external field are small and it is (iii) global and (iv) homogeneous. These four are in general violated for the reaction of a collective to a predator.

- **Equilibrium state**: We consider an active system and therefore per definition a non-equilibrium system. The agents dissipate constantly energy (no conservation of momentum) but, due to an unspecified energy source, keep their preferred speed, i.e. the system is out of thermal equilibrium.
- **Small changes** of an external field: In the context of a predator attack, the perturbing force is the flee-force of the agent. This flee-force can also be large and thus can dominate all other forces. Therefore, to compute the susceptibility by the linear approximation might not be justified.
- **Global field**: The global homogeneous field simplified the former analytical derivations of the susceptibility. However, the flee-force is neither global nor homogeneous. The flee-force acts only on agents that directly sense the predator. If we assume visual interactions with occlusion by conspecifics, but also with metric-, Voronoi-interaction and other local interaction types, the predator is per definition a local perturbation.
- **Homogeneous field**: The flee-force is in the simplest case a repulsion force and therefore inhomogeneous. However, close individuals have similar relative position with respect to the predator and therefore also a similar flee-force. Thus, locally the force can be approximated to be homogeneous.

The violation of the first assumption means that we can not ensure that the fluctuations in the order parameter represent the response of the system to an external field. However, as shown in Eq. S45 these fluctuations are analog with the sum over all pair correlations of velocity fluctuations. Furthermore, even if we assume that the susceptibility would represent the change of one non-equilibrium stationary state to another one due to an external field, it might be useless at the phase transition. Phase transitions are up to a certain degree analogous to bifurcations in dynamical systems, i.e. both mark the sudden emergence or extinction of steady states. Thus, as it is typical for bifurcations, also at phase transitions critical slowing down occurs. This means that the dynamic of the system slows down and the relaxation to the steady state takes longer the closer the system is to the phase transition. The attack of a predator is fast and the predator does not wait for the collective to reach a steady state to continue. This is an additional reason, with the other mentioned unmet assumptions, why the susceptibility should be considered with caution and why its link to optimal predator response is unclear.

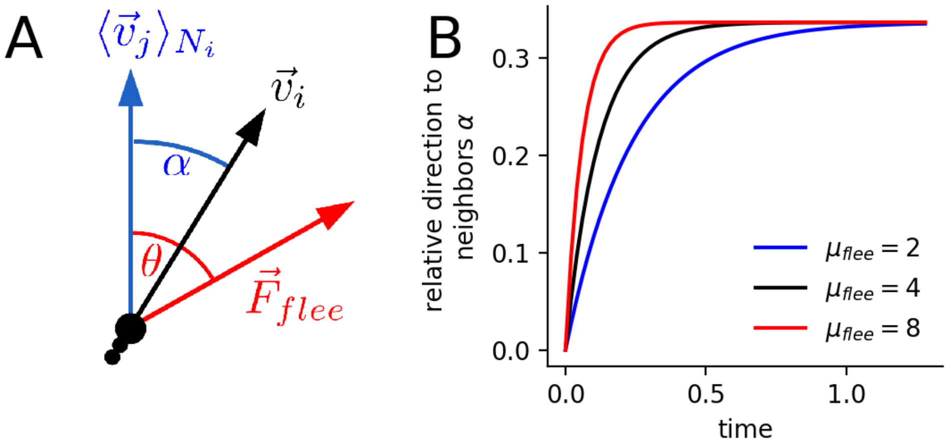

Figure S3: **Balancing social and private information via a directional compromise.** **A**: Illustration of angle-vector-relations for variables used in Eq. S47 and the following. The angle α is the angle between the mean velocity of neighbors $\langle \vec{v}_j \rangle_{\mathbb{N}_i}$ (blue arrow) and the velocity $\vec{v}_i$ of agent i (black arrow). The angle θ is the angle between the mean neighbor-velocity and the flee force $\vec{F}_{flee}$ (red arrow). **B**: Numerical-results of the relative direction to neighbors α using Eq. S47. The initial conditions is $\alpha = 0$, i.e. the focal agent is perfectly aligned with its neighbors. The angle between mean neighbor velocity and flee force is $\theta = \pi/2$.

VI Balancing social vs. direct predator information

We identified in the main text a possible explanation for the dependence of the evolutionary stable alignment strength on the flee strength as observed in the main text Fig. 4B. A prey can benefit from stronger alignment if it has no private information about the predators position. The benefit increases the faster the alignment and therefore should increase with alignment strength. But if the prey is fleeing already, i.e. it has private (direct) information on the predator position, than alignment to uninformed neighbors can hinder an escape. Therefore, we expect a balance between benefits and costs. In the following we will discuss a semi-analytical approximation which reproduces the observed linear dependence.

The costs to align with uninformed prey if the predator position is known can be viewed as a deviation from the flee direction, i.e. the prey relaxes to an effective flee direction which is the compromise between the mean direction of its neighbors and the flee direction Fig. S3.

We will use the following assumptions:

- i) highly ordered: all neighbors are perfectly aligned with each other.
- ii) strong forces: the acting forces are strong such that the agents equilibrate quickly in the direction of the force.
- iii) constant forces: the flee-angle and the heading of the neighbors are not changing.
- iv) no noise: this will enable us to solve the problem analytically.

Consequently the change of the direction-angle of Eq. S1b can be reformulated to

$$\frac{d\varphi_i}{dt} = \frac{1}{v}\left(F_{i,\varphi} + \sqrt{2D}\xi\right) \tag{S46a}$$

$$\approx \frac{1}{v}\left(F_{i,\varphi}\right) \tag{S46b}$$

$$\approx \frac{1}{v}\left(\mu_{flee}\hat{f}_{flee} + \mu_{alg}[\langle \vec{v} \rangle_{N_i} - \hat{e}_{r,i}]\right) \cdot \hat{e}_{\varphi,i}. \tag{S46c}$$

With $\langle \vec{v} \rangle_{N_i}$ being the mean velocity of all neighbors of agent i and $\hat{e}_{r,i}$ and $\hat{e}_{\varphi,i}$ are its heading and angular direction, respectively.

Without loss of generality we can permanently rotate the system such that $\varphi = 0, \forall t$ which simplifies the vector products since $\hat{e}_{r,i} = [1, 0] = \hat{e}_x$ and $\hat{e}_{\varphi,i} = [0, 1] = \hat{e}_y$. The angle α between $\vec{v}_i$ and $\langle \vec{v} \rangle_{N_i}$ behaves exactly opposite as φ (see Fig. S3A) and we describe its dynamics instead:

$$\frac{d\alpha}{dt} = -\frac{d\varphi}{dt} \tag{S47a}$$

$$\approx -\frac{1}{v}\left(\mu_{flee}\hat{f}_{flee} + \mu_{alg}[\langle \vec{v} \rangle_{N_i} - \hat{e}_x]\right) \cdot \hat{e}_y \tag{S47b}$$

$$\approx -\frac{1}{v}\left(\mu_{flee} f_{flee,y} + \mu_{alg}\langle \vec{v} \rangle_{N_i,y}\right). \tag{S47c}$$

With $f_{flee,y} = \sin(\theta - \alpha)$ and by assuming perfect order and unit speed the mean velocity of neighbors is $\langle \vec{v} \rangle_{N_i} = 1\begin{pmatrix}\cos(\alpha) \\ \sin(\alpha)\end{pmatrix}$. Therefore, the change of α simplifies to:

$$\frac{d\alpha}{dt} \approx -\frac{1}{v}\left(\mu_{flee}\sin(\alpha - \theta) + \mu_{alg}\sin\alpha\right) \tag{S48a}$$

$$\approx \mu_{flee}\sin(\theta - \alpha) - \mu_{alg}\sin\alpha. \tag{S48b}$$

The fixed points are, as a sanity check, computed for the extreme cases $\mu_{alg} \gg \mu_{flee}$ and $\mu_{flee} \gg \mu_{alg}$ which are $\alpha^\star = 0$ and $\alpha^\star = \theta$, respectively. There exist in general four fixed points from which only one fulfills the criteria $\alpha^\star/\theta \in [0, 1] \forall\, (\mu_{flee} > 0,\ \mu_{alg} > 0,\ 0 < \theta < \pi/2)$ which is:

$$\alpha^\star(\theta_s,\ \mu_{alg},\ \mu_{flee}) = \arccos\frac{\mu_{alg} + \mu_{flee}\cos\theta}{\sqrt{\mu_{alg}^2 + \mu_{flee}^2 + 2\mu_{alg}\mu_{flee}\cos\theta}}. \tag{S49}$$

Thus $\alpha^\star$ is the effective flee angle with respect to the mean direction of the neighbors. The closer it is to the flee angle θ the smaller the cost of being aligned given the knowledge of the predators position.

Now we assume that individuals evolve such that they maintain $\alpha^\star(\theta_s)$ with respect to a specific θ_s. Thus, if we know the equilibration point $\mu^\star_{alg,evo}(\mu_{flee,evo})$ for the specific flee strength that was used during the evolution $\mu_{flee,evo}$, we can compute the effective flee angle $\alpha^\star(\theta_s,\ \mu^\star_{alg,evo},\ \mu_{flee,evo}) = \alpha^\star(\theta_s)$. If we assume that agents evolve such that the balance between alignment benefit and cost, manifested in the effective flee angle, is kept constant, than we can predict the evolutionary stable state $\mu^\star_{alg}$ for a given flee strength by reformulating Eq. S49 to

$$\mu^\star_{alg} = \frac{\sin(\theta_s - \alpha^\star(\theta_s))}{\sin\alpha^\star(\theta_s)}\mu_{flee}. \tag{S50}$$

The term $\frac{\sin(\theta - \alpha^\star)}{\sin\alpha^\star}$ does not depend on θ_s which we confirmed numerically. Thus, the exact choice of θ_s is irrelevant and $\frac{\sin(\theta - \alpha^\star)}{\sin\alpha^\star}$ is only the slope which connects the origin and the one evolutionary stable state $(\mu^\star_{alg,evo}, \mu_{flee,evo})$ used to compute $\alpha^\star(\theta_s)$ as shown by the blue line in Fig. 4B.

Note that the equilibrium alignment strength $\mu^\star_{alg}$ above but close to the order transition is systematically lower than its predicted value, as seen for $\mu_{flee} \in \{2, 3, 4\}$ in Fig. 4B. This can be explained by a small signal due to the low flee strength, because the system relaxes faster the greater the flee strength μ_{flee} (see Fig. S3B). An alternative explanation is that the spatial selection due to strong self-sorting dominates at the transition. This explanation is also in agreement with the ESS for low flee strength ($\mu_{flee} = 0.5$) being identical to the one with no flee strength at all ($\mu_{flee} = 0$).

VII Robustness against modifications of the prey & predator dynamics and the selection mechanism

To ensure that our results are robust, we repeat the evolution (Fig. S4) with (i) modified prey properties, i.e. changing the angular diffusion coefficient and introducing variable speed and a blind angle, (ii) a changed predator behavior, i.e. its agility, and (iii) changes in the evolutionary selection mechanism, e.g. by an additional high-frontal-risk selection mechanism or by a prey capture during the simulation. Note that especially the additional high-frontal risk selection is of importance, because it introduces a heterogeneous environment which is assumed to be a general important condition for the evolution to criticality [4].

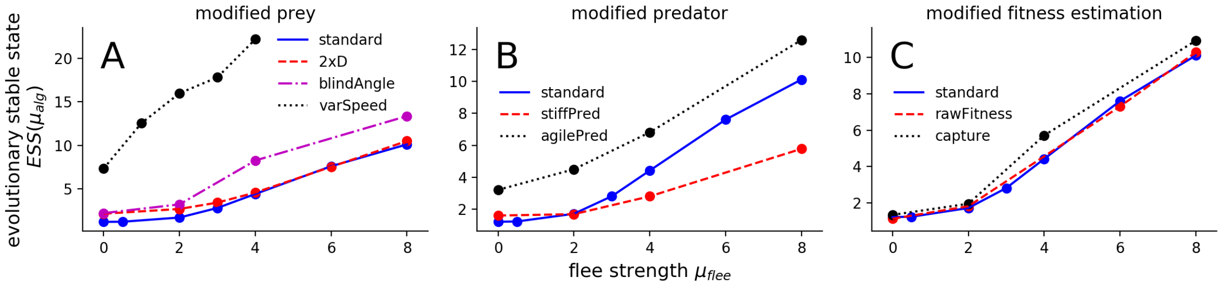

Figure S4: **Robustness analysis of evolution results.** Evolutionary stable states of the alignment strength are estimated from the fitness gradient for different flee strength under slight variations of simulations parameters or predator attack implementation. The standard scenario of the main text (blue line) is compared to (**A**:) a prey population with varying speed which can avoid the predator additionally by acceleration (black dotted line), a prey population with a angular diffusion coefficient which is doubled compared to the standard case (red dashed line), a prey population with a continuous blind angle (magenta dash dotted line), (**B**:) a less agile predator ("stiff") which turns less quick (black dotted line) and an more agile predator which turns quicker (red dashed line) than the predator in the standard case. (**C**:) a non-binarized fitness estimate (red dashed line) in which the prey's fitness is not defined by captures but by the accumulated probability of capture, a fitness estimate based on captures during the simulation (black dotted line),

VII.1 Prey modifications

The change in angular diffusion from $D = 0.5$ to $D = 1$ shifts the order-transition to a larger mean alignment strength of $\mu_{alg,c} \approx 1.6$ and therefore also increases the lower bound for the ESS which is visible in larger ESS for small flee strength (compare dashed red with blue line in Fig. S4A). For larger flee strength the results are nearly identical suggesting that the mechanism defining the ESS remains unchanged with respect to the standard scenario of the main text.

If the speed of the prey is not constant but can change according to social forces, the equations of motion (Eq. S1) change to

$$\frac{d\vec{r}_i}{dt} = \vec{v}_i \text{ with } \vec{v}_i = v_i[\cos\varphi_i, \sin\varphi_i] \tag{S51}$$

$$\frac{dv_i}{dt} = \beta(v_0 - v_i) + F_{i,v}(t) \tag{S52}$$

$$\frac{d\varphi_i(t)}{dt} = \frac{1}{v}\left(F_{i,\varphi}(t) + \sqrt{2D}\xi(t)\right) \tag{S53}$$

with $F_{i,v}(t) = \vec{F}_i \cdot \hat{e}_{h,i}$ as the projection of the social force of prey i on its heading direction $\hat{e}_{h,i}$ and β as the relaxation coefficient which is set in the following to $\beta = 4$. A value of $\beta = 4$ prevents the school to relax into a non-moving phase which exists for lower values of β [5]. In this non-moving state the speed of the prey would fluctuate around zero. Additionally, we set an upper bound for the prey's speed corresponding to eighty percent of the predators speed $v_{max} = 0.8v_p$. Non-fleeing prey ($\mu_{flee} = 0$) evolve to significant larger values compared to the standard scenario from the main text (compare dotted black with blue line in Fig. S4A). The ESS for non-fleeing prey ($\mu_{flee} = 0$) coincides with the zero-crossing of the front-sorting (Fig. S5). Not only is the ESS of the non-fleeing prey at larger values due to a different self-sorting but also is the ESS much more sensitive to changes in the flee strength (compare slope of dotted black with blue line Fig. S4A). This steeper increase is explainable with an additional social cue, the increased speed of fleeing neighbors, which is not present in the constant speed scenario and goes in hand with findings by Lemmasson et al. [6, 7].

We introduced an anisotropy of social interactions via a continuous angular preference: a focal agent i responds stronger to neighbors in front than to those at the side or behind. Mathematically, the preference depends on the relative angular position θ_{ij} of neighbor $j \in N_i$, which is the angle between the focal agents current velocity $\vec{v}_i$ and the relative position of the neighbor $\vec{r}_{ji}$). Following Calovi et al. [8], the preference decreases with θ_{ij}

$$\Omega_{ij} = 1 + \cos\theta_{ij}, \text{ with } \theta_{ij} = \angle(\vec{v}_i, \vec{r}_{ji}) \; . \tag{S54}$$

This corresponds to a continuous version of a blind angle. Thus, instead of computing the social forces by averaging over all Voronoi neighbors equally, a weighted average is performed to compute the alignment and distance regulating

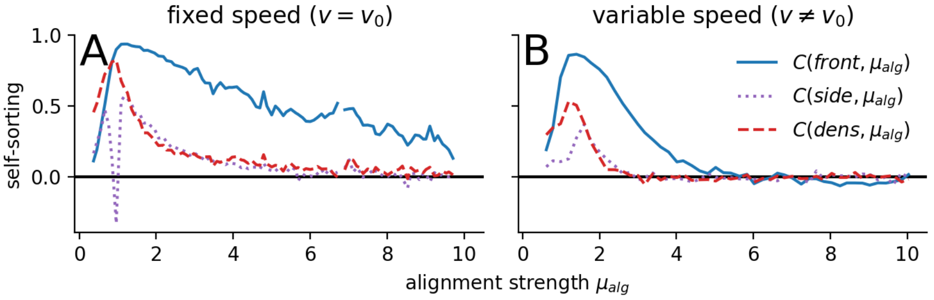

Figure S5: **Self-sorting with and without fixed speed.** Self-sorting quantified via the Pearson correlation between the individual alignment parameter μ_{alg} and the average relative position of the individuals (relative front-, side- or density-location as described in Sect. IV.1). **A**: If prey agents respond only by changing their direction but not their speed (fixed speed), self-sorting persists also in highly ordered regions. **B**: If prey agents can change their speed (variable speed), self-sorting vanishes for $\mu_{flee} \leq 6$.

force (Eqs. S2, S3). The weight is proportional to the angular preference Eq. S54. This modification leads to less averaging and therefore a higher sensitivity to noise which we measure via a decrease in the polarization for the same parameters as in the standard scenario. It effectively shifts the disorder-order transition to larger alignment strength (not shown). In agreement with the shifted disorder-order transition also the ESSs shift to larger alignment strength but the qualitative dependence on the flee strength and their location in the order regime are not altered in comparison to the standard scenario discussed in the main text (compare slope of dash dotted magenta with blue line Fig. S4A).

VII.2 Predator modifications

We repeated the simulations with (i) a less agile predator which turns slower and (ii) a more agile predator which turns faster compared to the predator considered in the main text. The different turning ability was implemented by modifying the pursuit strength μ_p to $\mu_p = 1$ for the less agile and to $\mu_p = 3$ for the more agile predator.
The effect of using the less agile predator is negligible for low flee-strength, probably because the order-disorder transition acts as lower bound for the ESS due to the explained maximum in assortative mixing and resulting subpopulation selection. However, for larger flee strength, e.g. $\mu_{flee} \in \{4, 8\}$ in Fig. S4B, the ESSs are lowered compared to the standard scenario in the main text. This can be explained by the missing feedback between the reaction of the prey and the trajectory of the predator: in the standard scenario the predator heads for the closest prey, thus if certain prey individuals are good at evading the predator, they have an additional fitness benefit because the predator pursues effectively primarily less well evading prey.
Consequently, the more agile predator increases the relative fitness benefit of better responding prey and thus amplifies the fitness gradient, which should push the ESS more in the already preferred parameter region. This is in fact observed (compare dotted black with blue line in Fig. S4B).
Despite the quantitative differences due to the predator modifications the general finding discussed in the main text remain unchanged, i.e. that the ESSs are in the ordered phase and increase with increasing flee-strength.

VII.3 Selection modification: Evolution in a heterogeneous environment

In the simulations prey are not captured but a fixed fraction of them with the largest accumulated probability of capture is declared as captured after the simulation. This means that no prey is removed during the simulation which reduces stochasticity of the fitness estimate but can be considered as unrealistic. If prey are removed during the simulation based on their current probability of capture and the predators attack rate, the evolution results remain unchanged (compare dotted black with blue line in Fig. S4C). Hereby the attack rate γ_a is adjusted at each generation g such that the mean capture rate $\langle\gamma_c\rangle$ matches the initially set attack rate $\gamma_a(g = 0)$:

$$\gamma_a(g + 1) = \gamma_a(g) * \frac{\gamma_a(0)}{\langle\gamma_c(g)\rangle}. \tag{S55}$$

This ensures a constant evolutionary pressure.

The attack rate parameter can be abandoned if the fitness is not estimated by the captures but by the negative accumulated probability of capture. This modification does not alter the ESS identified in the main text at all (compare dashed red with blue line in Fig. S4C).

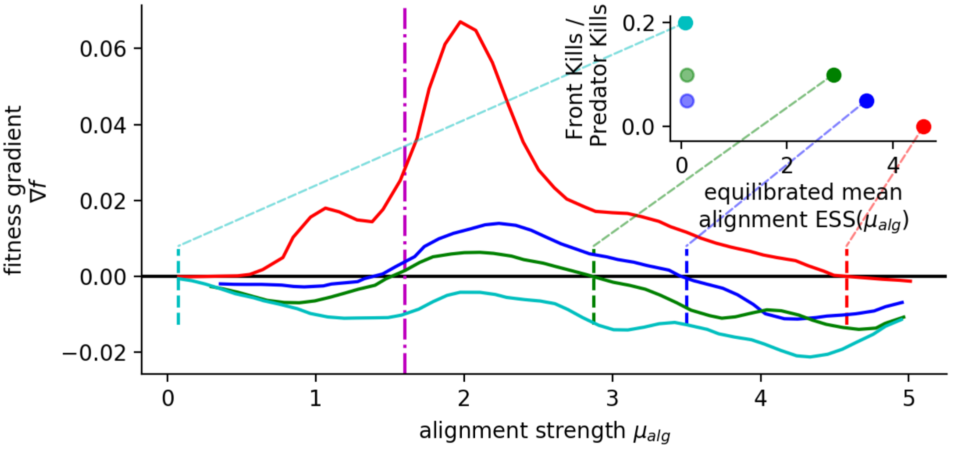

Figure S6: **Evolution in heterogeneous environments.** Fitness gradients for different relative strength of the frontal-risk selection with respect to the simultaneously active predator-selection. In the frontal-risk selection the most frontal individuals are declared as dead. The relative strength of the frontal-risk selection is defined by the ratio between agents killed at the front and by the predator, i.e. $(\text{Front Kills})/(\text{Pred. Kills}) \in [0,\ 0.05,\ 0.1,\ 0.2]$. The evolutionary stable state (ESS) is defined by the zero-crossing of the fitness gradient with negative slope marked by a vertical dashed line. However, the lower bound is an additional ESS if the fitness gradient stays negative close to it which is marked by shaded points in the inset. Parameters are identical to the former simulations apart from the angular diffusion coefficient which is increased to $D = 1$ increasing the order-transition to $\mu_{alg,c} \approx 1.6$ marked by vertical dash-dotted magenta line. The flee strength is $\mu_{flee} = 4$.

The chosen predator-prey interaction is set as general as possible, nevertheless reasonable alternatives exists and other environmental interactions, e.g. exploration and exploitation of food-sources, might simultaneously impact the fitness. We introduce an additional selection mechanisms which favors a disordered phase and creates thus a heterogeneous environment. The self-sorting for this model predicts that a high mortality of front individuals leads to a disordered state which we implement by declaring the most frontal prey as dead. This extra selection is equivalent with the observed high risk of being in the front in the presence of sit-and-wait predators [9]. Since the current transition is close to the lower boundary of the alignment parameter ($\min(\mu_{alg}) = 0$), we set the transition at larger values, i.e. at $\mu_{alg,c} \approx 1.6$, by increasing the angular diffusion to $D = 1$ (ensuring that fluctuations allow equilibration in the disordered regime).

The ESS with respect to alignment decreases with increasing weight on the frontal-risk selection (Fig. S6) which seems to be not surprising; however, in a similar study individuals evolved to criticality if exposed to a diverse environment [4]. In fact the transition acts here as a fitness valley, marked by a zero-crossing of the fitness gradient with positive slope, causing multiple local optima (inset in Fig. S6), which only vanish if one of the selection mechanisms dominates.

References

[1] P. Romanczuk and L. Schimansky-Geier, “Brownian Motion with Active Fluctuations,” *Physical Review Letters*, vol. 106, p. 230601, jun 2011.

[2] U. M. B. Marconi, A. Puglisi, L. Rondoni, and A. Vulpiani, “Fluctuation-dissipation: Response theory in statistical physics,” *Physics Reports*, vol. 461, no. 4-6, pp. 111–195, 2008.

[3] A. Sarracino and A. Vulpiani, “On the fluctuation-dissipation relation in non-equilibrium and non-Hamiltonian systems,” *Chaos An Interdiscip. J. Nonlinear Sci.*, vol. 29, p. 083132, aug 2019.

[4] J. Hidalgo, J. Grilli, S. Suweis, M. A. Munoz, J. R. Banavar, and A. Maritan, “Information-based fitness and the emergence of criticality in living systems,” *Proceedings of the National Academy of Sciences*, vol. 111, pp. 10095–10100, jul 2014.

[5] R. Großmann, L. Schimansky-Geier, and P. Romanczuk, “Active Brownian particles with velocity-alignment and active fluctuations,” *New Journal of Physics*, vol. 14, apr 2012.

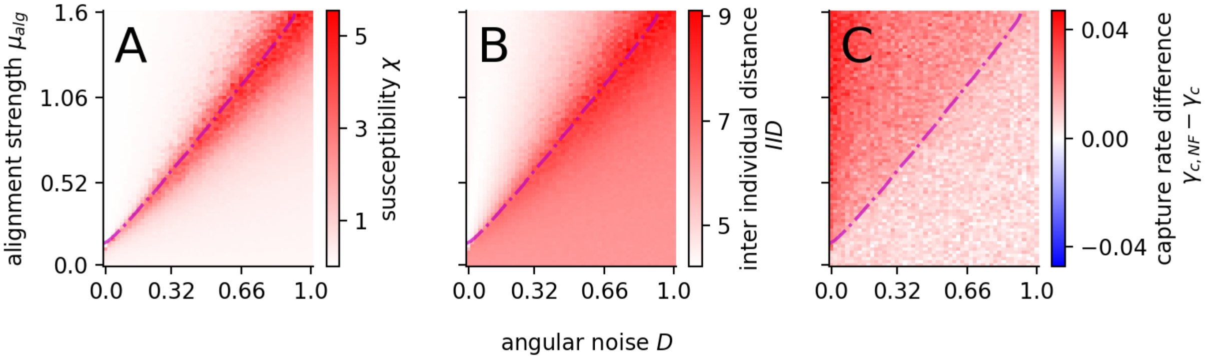

Figure S7: **Enlarged insets from main text Fig. 2.** The susceptibility χ (**A**), inter individual distance IID (**B**) and difference in capture rate between non-fleeing and fleeing individuals $_{c,NF} - \gamma_c$ (**C**). All measures are shown with colorbars, which were omitted for clarity in Fig. 2.

[6] B. H. Lemasson, J. J. Anderson, and R. A. Goodwin, "Collective motion in animal groups from a neurobiological perspective: The adaptive benefits of dynamic sensory loads and selective attention," *Journal of Theoretical Biology*, vol. 261, no. 4, pp. 501–510, 2009.

[7] B. H. Lemasson, J. J. Anderson, and R. A. Goodwin, "Motion-guided attention promotes adaptive communications during social navigation.," *Proceedings. Biological sciences / The Royal Society*, vol. 280, no. 1754, p. 20122003, 2013.

[8] D. S. Calovi, U. Lopez, S. Ngo, C. Sire, H. Chaté, and G. Theraulaz, "Swarming, schooling, milling: phase diagram of a data-driven fish school model," *New J. Phys.*, vol. 16, p. 015026, jan 2014.

[9] D. Bumann, D. Rubenstein, and J. Krause, "Mortality Risk of Spatial Positions in Animal Groups: the Danger of Being in the Front," *Behaviour*, vol. 134, no. 13-14, pp. 1063–1076, 1997.

S Videos

S1 Video

Animation of nine simulations. The red line are the past- and the empty red circle is the current center of mass of the collective. Animations in the same column are samples of the same parameter configuration. The columns differ in the alignment strength $\mu_{alg} = [0, 1, 2]$ indicated at the top. The remaining parameters are identical to the ones used in the main text (listed in Tab. S1).

S2 Video

Same as S1 Video but with a predator attacking the collective.

S3 Video

Attack simulation on non- and fleeing prey. The left panel shows only the fleeing prey, the right the non-fleeing prey, and the center shows both. The color-code is black=fleeing prey, blue=non-fleeing prey, red=predator attacking fleeing prey, green=predator attacking non-fleeing prey. Parameters are identical to the ones used in the main text (listed in Tab. S1).

S4 Video

Same as S2 Video but with other alignment parameters $\mu_{alg} = [2, 3, 4]$.

S5 Video

Animation of nine attack simulations with variable prey speed. Same as S2 but with preys that are able to accelerate according to the current force. The equations of motions for the prey with variable speed are defined in Sect. VII.
