# Supplementary figures and images for: Collective predator evasion: Putting the criticality hypothesis to the test
Source: PLoS Comput Biol. 2021 Mar 15;17(3):e1008832. doi: 10.1371/journal.pcbi.1008832 (PMC7993868; doi:10.1371/journal.pcbi.1008832)

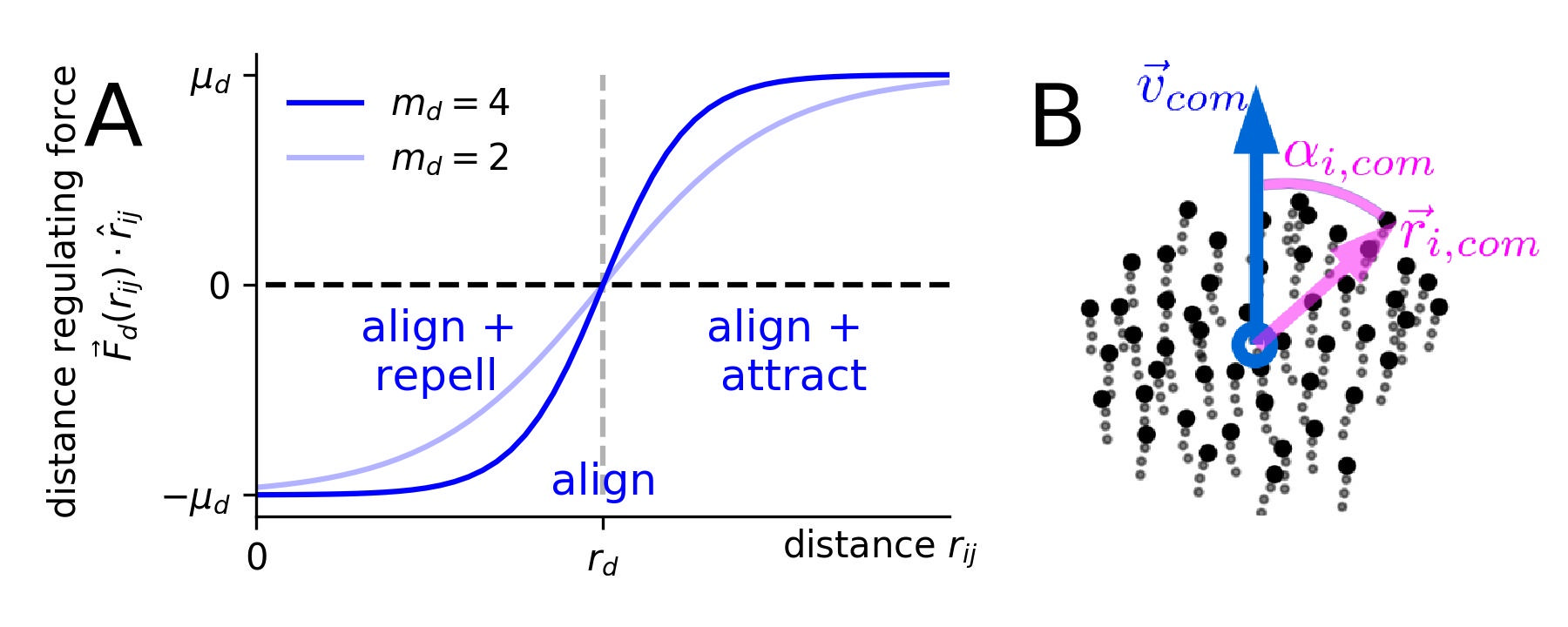

Supplement: S1 Fig — A: Distance regulating force F→d(rij) between agents i and j projected on the separation direction r^ji=r→j-r→i|r→j-r→i|. The force equals zero at the preferred distance rd = 1 and is displayed for a distance regulating force steepness md = 2 (used in the simulations) and md = 4. B: Relative polar coordinates of an agent i with respect to the center of mass r→com of the school (bluecircle) and to the average velocity of the school v→com (bluearrow). The angle αi,com (magentaarc) between the school velocity and the agents i current position r→i,com (magentaarrow) and the distance to the center of mass |r→i,com| define the position in this relative coordinate system. (TIF) [file pcbi.1008832.s002.tif]
