# Supplementary material for: Collective predator evasion: Putting the criticality hypothesis to the test
Source: PLoS Comput Biol. 2021 Mar 15;17(3):e1008832. doi: 10.1371/journal.pcbi.1008832 (PMC7993868; doi:10.1371/journal.pcbi.1008832)
Supplement: S1 Table — Time and space have been rescaled to dimensionless units by setting, without loss of generality, the prey speed v0 and preferred distance rd to 1. All length scales are thus measured in units of rd, and all time scales in terms of time needed to move the distance rd. Note that the flee strength μflee is strictly speaking a predator-prey parameter which reduces the prey-only parameters to four. (PDF) [file pcbi.1008832.s014.pdf]

	parameter	symbol	value
prey	angular diffusion	D	0.5
	alignment strength	θ_{alg}	evolves
	distance strength	θ_{d}	2
	distance steepness	m_{d}	2
	(distance preferred)	r_{d}	1
	(speed)	v_0	1
	f ee strength	θ_{flee}	4
predator	speed	v_{p}	2
	pursuit strength	θ_{p}	2
	attack rate	γ_{a}	1/3
	catch radius	r_{catch}	3
simul.	number of agents	N	400
	time step	dt	0.02
	equilibration time	T_{eq}	200
	simulation time	T_{simu}	120
	mutation rate	γ_{m}	0.8
	mutation strength	σ_{m}	0.075
